# Long-term clinical and radiological trajectories in *ANO5*-related myopathies highlight muscle MRI as a predictor of disease progression

**DOI:** 10.1007/s00415-026-13805-1

**Published:** 2026-04-15

**Authors:** Karolina Aragon-Gawinska, Pilar Martí, Inmaculada Azorín, Rafael Sivera, Elena Aller, Inmaculada Pitarch, Carlos Benlloch, María Tárrega, Rosa Vilar, Carmina Díaz, Rafael Sánchez-Roy, Fernando Más-Estellés, Marina Frasquet, Juan F. Vázquez-Costa, Mar Otero-Borell, Roger Vilchez, Juan J. Vilchez, Teresa Sevilla, Nuria Muelas

**Affiliations:** 1 Neuromuscular Diseases Unit, Neurology Department, Hospital Universitari I Politècnic La Fe, Member of the European Reference Network for Rare Neuromuscular Diseases (ERN EURO-NMD), Avenida de Fernando Abril Martorell, 106, 46026 Valencia, Spain; 2 Neuromuscular and Ataxias Research Group, Instituto de Investigación Sanitaria La Fe, Valencia, Spain; 3 Centro de Investigación Biomédica en Red de Enfermedades Raras (CIBERER), U763 Valencia, Spain; 4 Department of Medicine, Universidad CEU-Cardenal Herrera, Moncada, Valencia, Spain; 5 Cellular, Molecular and Genomics Biomedicine Group, Instituto de Investigación Sanitaria La Fe, Valencia, Spain; 6 Department of Genetics, Hospital Universitari I Politècnic La Fe, Valencia, Spain; 7 Department of Pediatric Neurology, Hospital Universitari I Politècnic La Fe, Valencia, Spain; 8 Department of Clinical Neurophysiology, Hospital Universitari I Politècnic La Fe, Valencia, Spain; 9 Department of Neurology, Hospital General de Castellon, Castellon, Spain; 10 Department of Neurology, Hospital General Universitario Doctor Balmis—ISABIAL, Alicante, Spain; 11 Department of Neurology, Hospital Arnau de Villanova-Llíria, Valencia, Spain; 12 Ascires, Neurorradiology Section, Área Clínica de Imagen Médica, Hospital Universitari I Politècnic La Fe, Valencia, Spain; 13 Department of Medicine, Universitat de València, Valencia, Spain

**Keywords:** Anoctamin, ANO5, Limb-girdle muscular dystrophies, Distal myopathies, Magnetic resonance imaging

## Abstract

**Background and objectives:**

Anoctamin-5 gene (ANO5)-related myopathies are clinically heterogeneous, and predictors of progression remain poorly defined. This study aims to characterize their long-term clinical and radiological evolution and identify factors influencing disease severity.

**Methods:**

We conducted a retrospective observational study of patients with anoctaminopathies in the Valencian Community (Spain). Demographic, clinical, genetic, ancillary tests, and serial muscle MRI data were analyzed using univariate and Bayesian multivariate models.

**Results:**

Thirty patients (21 males) from 23 families were included. Median disease duration was 13.5 years, and follow-up was 7 years (range 3–31). Median age at first assessment was 43.5 years (range 9–78). Most patients presented with pseudometabolic myopathy (47%) or asymptomatic hyperCKemia (37%), while 16% had LGMD-R12/MMD3. Nearly one-third of symptomatic patients had a documented asymptomatic hyperCKemia period (median 5.5 years). At last assessment, 27% had LGMD-R12/MMD3, 43% had pseudometabolic myopathy, and 30% remained asymptomatic. The most frequent variants were c.191dupA (35%) and c.692G > T (22%). CK levels, biopsy and EMG findings did not distinguish phenotypes. Baseline MRI revealed fat infiltration and STIR hyperintensities in most patients across all phenotypes. Muscle involvement progressed in 95% of serial scans. STIR hyperintensities preceded fat replacement in approximately 50% of muscles. More severe phenotypes correlated with older age, loss-of-function variants, and higher MRI T1 scores. Estimated prevalence was 1.8/100,000 in our region.

**Discussion:**

*ANO5*-related myopathies show progressive muscle involvement regardless of phenotype. Muscle MRI analysis provides valuable outcome measures for monitoring and future clinical trials, with STIR hyperintensities as a sensitive biomarker of disease activity.

**Supplementary Information:**

The online version contains supplementary material available at 10.1007/s00415-026-13805-1.

## Introduction

Anoctaminopathies are muscle disorders characterized by variable clinical presentations resulting from biallelic variants in the anoctamin-5 (*ANO5*) gene. Since their initial description in 2010 [1], these disorders have emerged as significant contributors to muscular disorders, ranging from asymptomatic or paucisymptomatic hyperCKemia to proximal or distal myopathies.

Typically, *ANO5*-related myopathies are adult-onset myopathies that primarily affect the posterior compartment of the lower limbs, often with asymmetrical involvement and slowly progressive course [2, 3]. While muscle hypertrophy is common in early stages, localized atrophy tends to develop over time, particularly in the quadriceps and biceps [3]. Most patients retain the ability to walk independently well into late adulthood [2]. Although respiratory involvement is rare [2], cardiac involvement, particularly left ventricular dysfunction or dilated cardiomyopathy is a possible complication in up to 30% of patients [4, 5].

Biallelic *ANO5* variants have variable presentations that can be categorized into four clinical phenotypes: limb-girdle dystrophy type R12 (LGMD R12, formerly known as LGMD2L), Miyoshi distal muscular dystrophy type 3 (MMD3), asymptomatic hyperCKemia, and paucisymptomatic hyperCKemia/pseudometabolic myopathy, which presents as myalgia, exercise intolerance, or recurrent rhabdomyolysis [3]. Notably, some individuals may remain asymptomatic for years, while others may progress to LGMD-R12 or MMD3 phenotypes [2, 6, 7]. An overlap between phenotypes has been observed and it has been suggested that a clear division into distal and proximal forms may not be justified [2, 7].

Over 120 different deleterious variants have been reported, the most common being c.191dupA (considered a founder mutation in Northern Europe) [8] and c.1898 + G > A [3]. Genotype–phenotype correlations remain limited [3], though biallelic loss-of-function (LOF) variants may predict more severe progression [2]. The prevalence of *ANO5*-related myopathies varies by geographic region, with the highest rates reported in Northern European countries (0.26–2 per 100,000) [2, 3, 5, 9–11] where LGMD-R12 represents a leading subtype, yet prevalence remains unknown in other countries, including Spain. Additionally, a clear male predominance is widely observed across cohorts, ranging from 2:1 to 4:1 [3, 9, 12].

Magnetic resonance imaging (MRI) is a powerful tool for diagnosing and characterizing muscle disorders [13, 14]. A characteristic pattern of muscle involvement has been described in *ANO5*-related myopathies, even in asymptomatic patients [6, 8, 15–18], with preferential involvement of the gastrocnemius medialis (GM), adductor magnus, semimembranosus, and semitendinosus muscles, often exhibiting asymmetry between sides [17–20]. Hyperintensities in short-tau inversion recovery (STIR) sequences are common early findings and may precede fatty degeneration[7]. However, data of clinical and radiological progression in *ANO5*-related myopathies are restricted to small cohorts or individual cases [2, 6, 9, 12, 19] and factors influencing disease progression remain poorly defined.

This study aims to characterize the natural history of a cohort of anoctaminopathies from a neuromuscular reference center, including radiological data, to evaluate predictors of disease severity, and estimate prevalence in the Valencian Community (Spain).

## Methods

This study, conducted at the Neuromuscular Reference Centre of Hospital UiP La Fe (Valencia, Spain), included 30 patients who met the following inclusion criteria: 1) confirmed genetic diagnosis based on detection of biallelic pathogenic variants in *ANO5* and 2) regular follow-up of greater than 3 years. All recruited patients gave their informed consent for participation. The research protocols have been approved by the Ethics Committee of the IIS La Fe (2022–701-1). The study was performed and reported in accordance with the Strengthening the Reporting of Observational Studies in Epidemiology (STROBE) guidelines for cohort studies [21].

Data were collected from hospital medical records. Demographic data (gender, ethnicity, and age), family history and genetic studies were reviewed. Genetic variants were classified as missense, frameshift, nonsense, or intronic; frameshift and nonsense variants were marked as LOF variants.

Clinical and ancillary test data were reviewed at first and follow-up visits. Symptoms (weakness, myalgia, exercise intolerance, or fatigue) and signs from the neurological examination (presence and distribution of muscle weakness, hypertrophy, and atrophy; asymmetry of muscle involvement) were recorded. Ancillary tests, performed by standard methods [22, 23], were reviewed, including serum CK values (minimum, maximum, and mean), muscle biopsies (classified as dystrophic or myopathic), electromyography (EMG, classified as normal or myopathic) and muscle MRI. Potential respiratory and cardiac involvement was assessed by evaluating the presence of symptoms and the results of standard respiratory function and cardiac tests performed.

Patients were classified into one of the following phenotypes according to their presentation: asymptomatic hyperCKemia (no symptoms and normal neurological examination), *pseudometabolic phenotype* (symptoms such as myalgia or exercise intolerance, but no weakness on examination), and *LGMD-R12/MMD3 phenotype* (when weakness was detected, regardless of its distribution as we considered both proximal and distal involvement as a continuum). Phenotypic changes were assessed during follow-up and the age at which they occurred was recorded.

### MRI analysis

Most MRI assessments were performed using a whole-body protocol (WBMRI) (20), which included T1-weighted and STIR sequences. Patients’ phenotypes and ages at MRI were recorded. Presence of intramuscular fat in muscles from three lower-body compartments (10 muscles of the lower legs, 12 of the thighs and 3 of the pelvis) in both lower limbs was scored in T1-weighted images using the semiquantitative scale of Mercuri modified by Fischer (30) (0–no changes, 1–traces of fatty infiltration with large areas of unaffected muscle, 2–clear fatty infiltration affecting < 50% of the muscle, 3–clear fatty infiltration affecting > 50% of the muscle, 4–practically all muscle area covered by fatty infiltration). We calculated a combined score by compartment and a total lower-body (TLB) score summing all three compartment scores. We also analyzed the number of muscles showing any grade of fat infiltration in each compartment. The annual rates of progression of the TLB score and number of affected muscles were calculated as the difference between each of these two parameters comparing the last and first MRIs, divided by the time interval in years between scans. Infiltration of lumbar paraspinal muscles was assessed in a bimodal way (yes/no). The presence of STIR hyperintensities was assessed (0 = absent, 1 = present) and correlated with changes in the Mercuri score between consecutive MRIs for each muscle. Score values were presented as median and inter-quartile range [q1 q3].

### Statistical analysis

The following variables were analyzed: phenotype (asymptomatic, pseudometabolic, LGMD-R12/MMD3); presence of LOF variants; age; gender; MRI variables (compartment and TLB scores, number of affected muscles, annual progression rate, and STIR hyperintensities); CK levels; muscle biopsy findings; and EMG results.

Univariate analysis was performed using Fisher’s exact test for categorical variables and the Kruskal–Wallis test for continuous variables at baseline and at the last visit. Correlations were assessed with Spearman’s rank test.

To identify factors influencing clinical phenotype, we apply a multivariate Bayesian ordinal regression analysis using a mixed-effects model that incorporates all longitudinal data points (baseline, intermediate, and last visit). After adjusting for gender, age at MRI, and genotype, several predictors including T1 scores and muscle involvement were evaluated. The TLB score was selected as the primary predictor in the final model, as it provided the strongest balance between predictive accuracy and the patient’s phenotype. The model showed appropriate statistical convergence and reliability.

## Results

Thirty individuals were included of which 21 were males. They belonged to 23 families with 5 pairs of siblings and 2 pairs of relatives. All of them lived in the Valencian Community (Spain) and were Caucasian, except for two siblings of Roma origin. Based on these data, the minimum prevalence of *ANO5*-related myopathies in our region was calculated as 1.8 per 100,000 individuals. Partial data from ten patients has been previously reported[6].

### Descriptive data

Table 1 shows the characteristics of the cohort.

**Table 1:** Descriptive data of the cohort

	Males,* n* = 21	Females,* n* = 9	All,* n* = 30
Age at onset total cohort, *y:* median [Q1,Q3] (range)	29 [22, 40] (7–66)	38 [35, 43] (29–73)	34 [24, 40.8] (7–73)
Age at onset per phenotype*, y*			
-Asymptomatic HCK,* n*	23 [15, 40.8] (7–66),* n* = *10*	45 [41.8, 53.5] (38–73)* n* = *4*	39 [16.8, 43.8] (7–73)* n* = *14*
-Pseudometabolic,* n*	35 [29, 38.8] (13–52)* n* = *10*	36 [31.2, 37.8] (29–50)* n* = *6*	36 [29.8, 38.2] (13–52)* n* = *16*
-LGMD-R12/MMD3,* n*	41 [39, 50] (24–69)* n* = *7*	75,* n* = *1*	43 [39.5, 58.5] (24–75)* n* = *8*
Median age at 1 st visit,* y*	38 [26, 44] (9–70)	47 [46, 55] (32–78)	43.5 [30, 47](9–78)
Phenotype at 1 st visit,* n (%)*			
-Asymptomatic	9 (43%)	2 (22%)	11 (38%)
-Pseudometabolic	8 (38%)	6 (67%)	14 (46%)
-LGMD-R12/MMD3	4 (19%)	1 (11%)	5 (16%)
Median follow-up,* y*	8 [6, 14] (3–31)	5 [4, 7] (3–15)	7 [5, 12.5] (3–31)
Median disease duration,* y*	13 [9, 19] (8–43)	14 [10, 20] (7–35)	13.5 [9.2, 19.8] (7–43)
Median age at last visit,* y*	46 [41, 55] (16–77)	57 [50, 65] (36–83)	49 [42.2, 56.8] (16–83)
Phenotype at last visit,* n (%)*			
-Asymptomatic	7 (33%)	2 (22%)	9 (30%)
-Pseudometabolic	7 (33%)	6 (67%)	13 (40%)
-LGMD-R12/MMD3	7 (33%)	1 (11%)	8 (30%)
Documented asymptomatic HCK period,* y*	13 [8.2, 19.8] (3–31) *n* = *10*	5 [2.8, 10.5] (2–21) *n* = *4*	10.5 [7.2, 19.8] (2–31)* n* = *14*
Serum CK levels (U/L)			
-Mean	3603 [1761.5, 5750] (857–7580)	916 [776, 1502.5] (355–2426)	2342 [1263, 4583.9] (355–7580)
-Minimum	948 [550, 1895] (312–3879)	362 [300, 690] (149–1283)	780.5 [390.8, 1392.5] (149–3879)
-Maximum	6276 [3211,10351] (1328–13,000)	1470 [1147, 2337] (400–3568)	3878 [1689.5, 8362.5] (400–13000)

Data are presented as median [Q1,Q3] (range);* n* number*, 1st* first,* y* years, *HCK* hyperCKemia

The median age at first visit was 43.5 years (range: 9–78), and was higher in females than in males. At first visit, 16% of the cohort presented with LGMD-R12/MMD3, while 47% exhibited pseudometabolic symptoms, and 37% were asymptomatic. Half of the cohort was referred after the detection of persistent hyperCKemia. Of those, one presented with the LGMD-R12/MMD3 phenotype, four complained of pseudometabolic manifestations and the remaining 11 were fully asymptomatic. Four other patients in the cohort were referred to rule out an inflammatory myopathy, including two who were on statin therapy. Patients were followed for a median of 7 years (range: 3–31) and had a median known disease duration of 13.5 years (range: 5–43). At last assessment, 27% of patients had muscle weakness while 43% complained of pseudometabolic symptoms and 30% remained asymptomatic. The LGMD-R12/MMD3 phenotype had a later age of onset compared with pseudometabolic and asymptomatic hyperCKemia phenotypes (median 43 vs. 36 and 39, respectively).

### Clinical features

Main clinical findings of our cohort are shown in Table 2.

**Table 2:** Clinical and ancillary features

Patient	P01	P02	P03	P04	P05	P06	P07	P08	P09	P10	P11	P12	P13	P14	P15	P16	P17	P18	P19	P20	P21	P22	P23	P24	P25	P26	P27	P28	P29	P30
Gender	M	M	M	M	M	M	M	F	M	F	M	M	F	F	M	M	M	M	F	M	M	M	F	M	M	M	F	F	F	M
Age at 1 st visit, y	31	46	69	51	13	29	25	59	43	49	40	39	31	46	15	20	44	20	43	43	30	25	46	9	45	38	46	55	79	47
Phenotype at 1 st visit, y	W	W	W	W	P	P	P	P	P	P	P	P	P	P	A	A	A	A	A	A	A	A	A	A	A	P	P	P	W	P
Age at last visit, y	56	67	77	56	43	40	35	65	49	57	46	46	36	49	44	43	48	41	59	57	37	29	50	16	53	42	51	66	83	55
Phenotype at last visit	W	W	W	W	W	P	P	P	P	P	P	P	P	P	A	A	A	W	A	W	A	A	A	A	A	P	P	P	W	P
Time FU, y	25	21	8	5	30	11	10	6	6	8	6	7	5	3	29	23	4	21	16	14	7	4	4	7	8	4	5	11	4	8
Period of asymptomatic HCK, y	un	21	3	un	un	un	un	un	un	un	un	un	un	un	31	28	8	16	21	8	8	14	7	9	12	un	un	3	2	un
Age at onset of pseudometabolic manifestation, y	–	–	–	–	13	28	24 *	30	41	37	32	37	29	35	–	–	–	38	–	52	–	–	–	–	–	33	38	50	–	39 *
Age at onset of LGMD–R12/MMD3, y	24	45	69	41	38	–	–	–	–	–	–	–	–	–	–	–	–	40	–	55	–	–	–	–	–	–	–	–	75	–
Weakness distribution in lower limbs	DP	DP	DP	DP	DP	–	–	–	–	–	–	–	–	–	–	–	–	P	–	D	–	–	–	–	–	–	–	–	AP	–
Weakness distribution in upper limbs	P	P	–	P	P	–	–	–	–	–	–	–	–	–	–	–	–	P	–	–	–	–	–	–	–	–	–	–	P	–
Walking aids (age)	46	50	–	–	–	–	–	–	–	–	–	–	–	–	–	–	–	–	–	–	–	–	–	–	–	–	–	–	–	–
Muscle hypertrophy	–	+	+	+	+	+	+	–	+	–	+	+	+	–	+	+	–	–	+	+	+	+	+	–	–	–	–	–	–	+
Muscle atrophy at 1 st visit	+	+	+	–	–	–	–	–	–	–	–	–	–	–	–	–	–	–	–	–	–	–	–	–	–	–	–	–	+	–
Muscle atrophy at FU	+	+	+	+	+	–	–	–	–	–	+	–	–	–	–	–	–	+	+	+	–	–	–	–	–	–	–	–	+	–
Asymmetry at 1 st visit	+	–	+	–	+	–	+	–	+	–	–	–	–	–	–	–	–	+	–	–	–	–	–	–	–	–	–	–	+	–
Asymmetry at FU	+	+	+	–	+	–	+	–	+	–	+	+	–	–	–	–	–	+	+	+	–	–	–	–	–	–	–	–	+	–
EMG	M	M	M	M	na	na	M	N	N	M	M	M	N	N	M	N	na	M	N	M	N	N	M	na	N	na	N	M	M	M
Biopsy	dys	dys	myo	dys	dys	NA	myo	myo	dys	dys	NA	dys	NA	NA	myo	myo	NA	myo	myo	NA	myo	NA	NA	NA	myo	myo	myo	myo	NA	myo
Cardiac findings (ECHO)	no	NA	IVSH	no	RVD	no	no	no	LVH	NA	no	IVSH	no	no	no	RVD	NA	NA	no	MI	no	no	NA	no	NA	no	NA	no	no	LVH
Respiratory involvement	–	–	–	OS	OS	–	–	–	–	–	–	–	–	–	–	–	–	–	–	–	–	–	–	–	–	–	–	–	–	–

M/F *1st* first, *FU* follow-up, Phenotype*: W* LGMD-R12/MMD3, *P* pseudometabolic phenotype, *A HCK* hyperckemia, *—rhabdomyolysis (P07 and P30), *un* unknown, Distribution of weakness/hypertrophy: *DP D* distal, *P* proximal, *AP* axial and proximal, *UL* upper limbs, *G* generalized, Biopsy findings: *dys* dystrophic, *myo* myopathic features, *EMG M* myopathic, *N* normal, *NA* not available, *ECHO* echocardiogram, *IVSH* interventricular septal hypertrophy, *RVD* right ventricle dilatation, *LVH* left ventricle hypertrophy, *MI* remodelling after myocardial infarction, *OS* obstructive sleep apnea syndrome

At first visit, among the five patients with LGMD-R12/MMD3, three exhibited disto-proximal weakness in lower limbs, waddling gait and difficulties with tiptoe and/or heel walking; one had proximal weakness in both upper and lower limbs, and one presented camptocormia due to prominent axial weakness. All were able to walk independently.

Over time, three additional patients developed weakness with heterogeneous distribution and variable age of onset (range: 38–55). Progression of muscle weakness was observed in all LGMD-R12/MMD3 patients, particularly in P01 and P02 requiring walking aids at ages 46 and 50, respectively. No patient was wheelchair-bound at last assessment.

Fourteen patients presented pseudometabolic manifestations, including myalgia and exercise intolerance. Two of them experienced episodes of rhabdomyolysis. During follow-up, most patients reported progression of symptoms that increasingly interfered with daily and work-related activities.

Muscle hypertrophy was observed in 60% of patients predominantly affecting the calves, while atrophy, detected in 13%, was restricted to patients with LGMD-R12/MMD3, affecting the lower legs (3 patients), quadriceps (1 patient) and biceps braquialis (1 patient). Asymmetric involvement was noted in 23% at first assessment and increased to 40% at last follow-up, mainly in LGMD-R12/MMD3. Over time, 30% of the cohort developed muscle atrophy, mainly in the lower legs, quadriceps and biceps. Two patients showed periscapular atrophy and scapular winging. Muscle atrophy affected all LGMD-R12/MMD3 patients but also occurred in one pseudometabolic and one asymptomatic individual.

### Phenotype evolution during follow-up

**Fig. 1:**
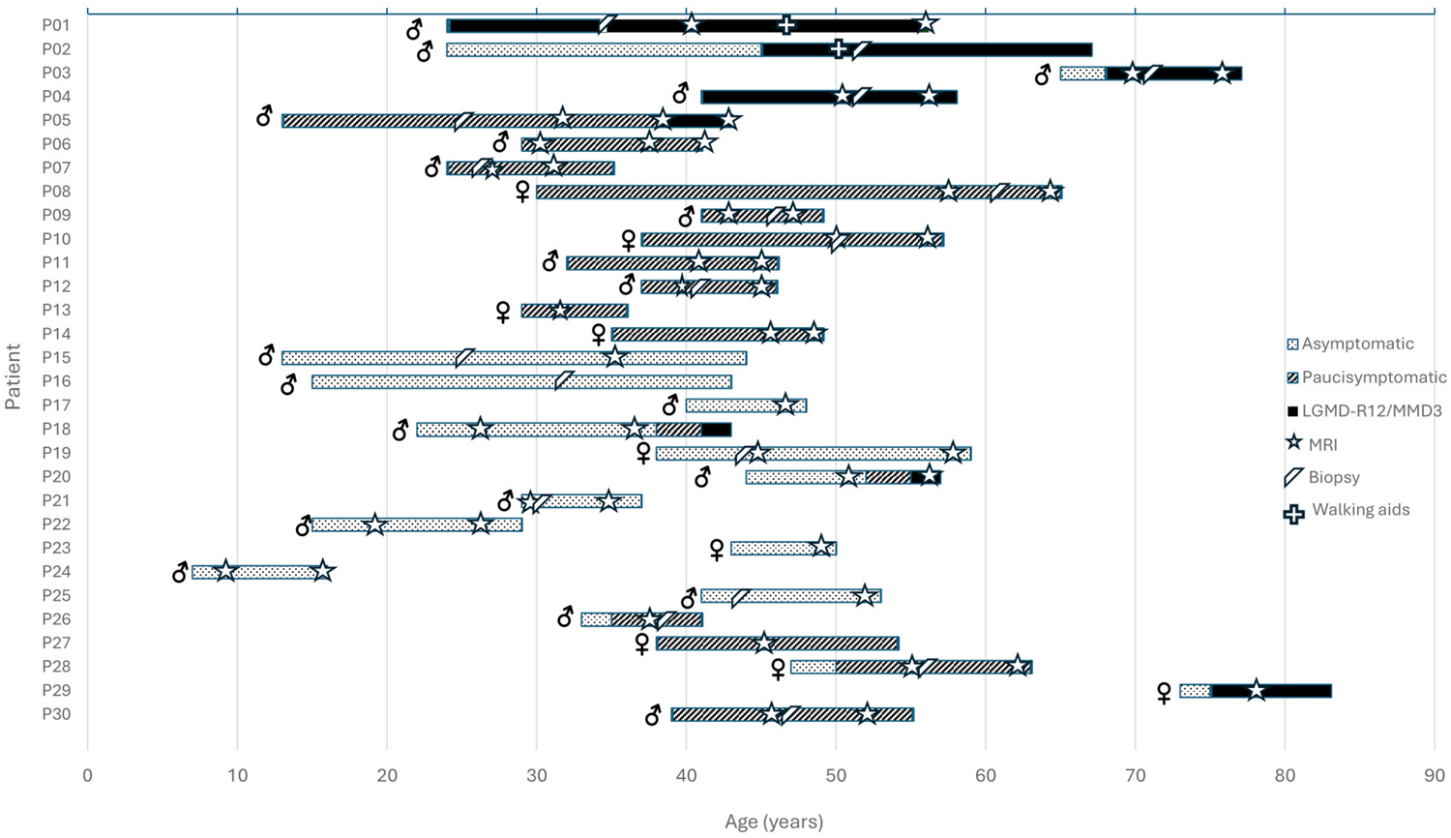
Phenotypic evolution of the cohort during follow-up. Evolution of the individuals’ clinical phenotypes during follow-up is represented. Symbols indicate age at MRI and at muscle biopsy, and first use of walking aids. Approximately 25% of the patients changed their phenotype during the follow-up period

**Fig. 2:**
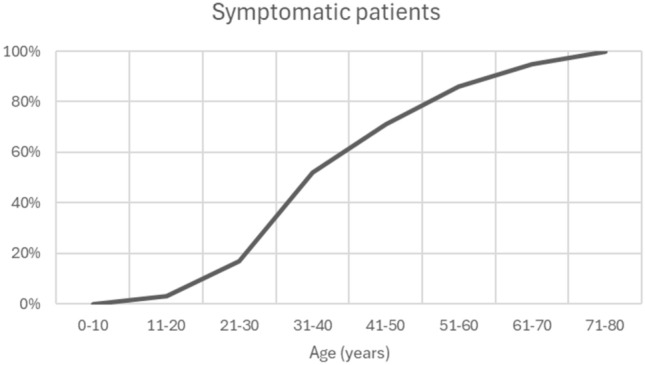
Proportion of symptomatic patients per life decade based on clinical status at last follow-up. Patients were considered asymptomatic until the documented onset of symptoms and symptomatic thereafter. Symptom prevalence increased progressively with age, showing the steepest rise between the fourth and sixth decades of life

The distribution of patients per phenotypes and their evolution during follow-up is represented in Fig. 1. Fourteen patients had a documented asymptomatic hyperCKemia period (median 10.5 years; range: 2–31). A period of pseudometabolic manifestations of variable duration (2–25 years) prior to the development of LGMD-R12/MMD3 phenotype was recognized in three patients, while five patients did not report any symptoms before developing muscle weakness. Three males changed their phenotype during follow-up: one paucisymptomatic patient developed weakness at age 38; two asymptomatic patients complained of pseudometabolic symptoms at age 38 and 52 and both developed weakness three years later. We observed that the percentage of symptomatic patients increased over life decades, especially between the 4th and 6th decade (Fig. 2).

### Genetic results

Fifteen variants in *ANO5* were identified, comprising four frameshift, eight missense, two nonsense and one non-coding variant (supplementary Table S1). Eleven variants were classified as pathogenic/likely pathogenic and four as variants of uncertain significance (VUS) according to the American College of Medical Genetics (ACMG). Five variants were novel in our cohort (four of them previously reported [6]); three were classified as VUS and predicted to be deleterious by in silico analysis: c.653A > G, c.950C > A, and c.2317A > G. The novel variants c.2339_2349del and c.2222C > G were classified as likely pathogenic. The c.155A > G variant, previously reported with conflicting clinical significance, was identified in compound heterozygosity in two unrelated patients, further supporting its pathogenicity.

The most frequent variant in the cohort was c.191dupA (16 individuals, five homozygous), followed by c.692G > T (12 patients, one homozygous).

Two families exhibited pseudodominant inheritance (father/daughter and aunt/nephew). These relatives shared one pathogenic variant and had a different variant in the other allele.

### Ancillary tests

Main results of complementary tests are shown in Tables 1 and 2. All patients showed elevated serum CK levels (median value 2342 U/L; range: 355–7580 U/L). CK levels fluctuated during the follow-up, and 20% of patients showed a decreasing trend in CK values.

Ten patients had hypertransaminasemia detected > 1 year before hyperCKemia, leading to an initial misdiagnosis of liver disorders.

EMG, performed in 25 patients, revealed myopathic changes in 60%, with spontaneous activity at rest in 36%, while 40% had normal findings.

Muscle biopsy was performed in 19 patients from deltoid (68%), GM (16%) and tibialis anterior (TA, 11%). Median age at biopsy was 43.5 years (range: 25–71). Dystrophic changes were observed in 39% of biopsies, while the remaining had unspecific myopathic features. The proportion of dystrophic features was highest if GM was biopsied (100%) compared with TA (50%) and deltoid (23%). Inflammatory infiltrates and muscle fibre necrosis were observed in two and three biopsies, respectively.

Cardiac assessments, available for 23 patients, detected structural abnormalities in seven male patients (30%): mild and non-progressive right ventricular dilation (*n* = 2), mild interventricular septal hypertrophy or mild concentric left ventricular hypertrophy (*n* = 4), and ventricular remodelling after myocardial infarction (*n* = 1). All patients maintained preserved systolic function, with ejection fractions (LVEF) remaining within normal limits. No patients required specific cardiological intervention. Respiratory function evaluations performed in seven patients did not reveal significant pulmonary restriction. Two patients were diagnosed with obstructive sleep apnea syndrome.

### Muscle MRI

MRI scans were available for analysis in 29 patients. Muscle imaging findings per patient and by grouping patients according to phenotype are detailed in supplementary Tables S2 and S3. The median age at first imaging was 43 years (range: 9–78 years). Longitudinal MRI data were available for 22 patients, with a median inter-study interval of 6.5 years (range: 3–16 years).

Intramuscular fat infiltration was detected in 24 patients (83%) at the first scan affecting the lower legs (75%) and pelvic muscles (68%), and less frequently, the thighs (55%). Isolated STIR hyperintensities without fat replacement were observed in two individuals, while the other three had normal MRI studies (aged between 19 and 45 years).

**Fig. 3:**
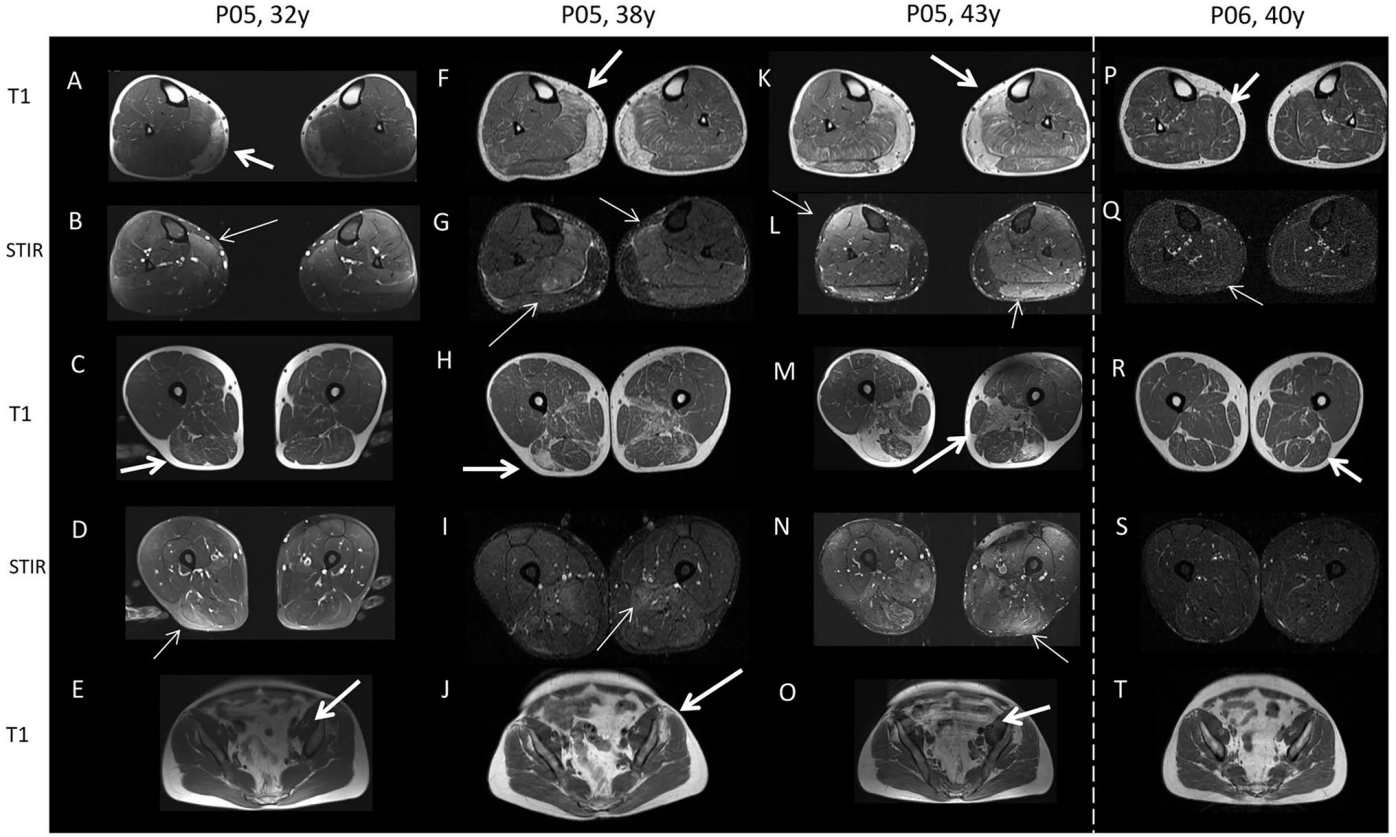
Longitudinal MRI data of the cohort. **A** Progression of the total lower-body (TLB) score between the first and last MRI. The dashed line represents the change from a pseudometabolic to LGMD-R12/MMD3 phenotype; dotted lines represent the change from asymptomatic to LGMD-R12/MMD3; the double line represents scores reduced by half for visibility purposes. **B** Number of muscles with and without STIR hyperintensities and their progression of fat infiltration across subsequent MRIs. **C** Progression of TLB and compartment-specific T1 scores. The left bar represents the first MRI, and the right bar the last available MRI. Colour coding: lower leg (dark grey), thigh (medium grey), pelvis (light grey). The total bar height represents the TLB score. Values for patient P01 were reduced by half for visibility purposes; *y* years, *M* male, *F* female

At baseline, the median TLB score of the cohort was 11.0 [4.0; 19.57]. Patients with LGMD-R12/MMD3 exhibited the highest scores in the three compartments and, consequently, the highest TLB scores compared with asymptomatic and pseudometabolic groups (Table S3). These differences were more pronounced between LGMD-R12/MMD3 and pseudometabolic individuals (*p* < 0.01), with the latter presenting the lowest scores. TLB scores in LGMD-R12/MMD3 differed significantly from those in asymptomatic individuals, although some overlap was observed (Fig. 3A). In contrast, TLB scores did not differ significantly between asymptomatic and pseudometabolic patients.

During follow-up, 95% of patients had progression of fat infiltration regardless of their phenotype (Fig. 3C). Significant differences in TLB score of LGMD-R12/MMD3 and pseudometabolic (*p* < 0.01) and asymptomatic groups (*p* < 0.05) persisted (Table S3).

The number of affected muscles followed a similar trend. At the first MRI, the LGMD-R12/MMD3 group had the highest number of affected muscles, followed by asymptomatic individuals, whereas the pseudometabolic group had fewer affected muscles across all compartments. The median number of affected muscles increased in the cohort during follow-up, mainly in lower legs and thighs, with the pelvis region remaining stable. The TLB score annual progression rate was higher in LGMD-R12/MMD3 phenotype (2.0 [2.0;2.4]) than in asymptomatic (1.1 [0.4;1.9]) and pseudometabolic (0.8 [0.7;1.3]) groups (*p* = *0.08)*, while the increase in the number of affected muscles per year was similar.

At baseline MRI the most affected muscles were GM (72%), gluteus minimus (66%) and soleus (55%) followed by adductor magnus (45%), semimembranosus and biceps femoris long head (41% each). The TA and vastii were affected only in more severe cases while rectus femoris, sartorius and gracilis were generally spared. Mild involvement of gluteus major and medius was observed in 14% of patients, who mainly presented the LGMD-R12/MMD3 phenotype. One patient had isolated gluteus minimus involvement, sparing the lower legs and thighs.

At follow-up MRI, an increase in fat infiltration was observed with a similar distribution to baseline but overall more widespread. The gluteus minimus became the most frequently affected muscle (86%), followed by GM (83%). Lumbar paraspinal muscle involvement, present in 17% of patients at first imaging, increased to 40% at follow-up.

STIR hyperintensities were observed in 76% of baseline scans and in 95% at follow-up MRI. They predominated in the lower leg muscles, affected the thighs in almost half of the patients, and were rare in the gluteii (5%). Progression of fat infiltration between consecutive scans occurred in nearly half of the muscles with prior STIR hyperintensities, compared to 15% of those without (*p* < 0.0001, Fig. 3B). Accordingly, 20% of the muscles that showed progression of fat infiltration had STIR hyperintensities on the previous MRI, compared with 5% of those without progression (*p* < 0.0001). Among muscles with STIR hyperintensities but no baseline fat infiltration, 25% showed progression, whereas involvement progressed in 65% of muscles displaying both STIR hyperintensities and fat infiltration.

### Correlation between variables, phenotypes and genotypes

#### *Age*

Age correlated with disease severity. Patients with LGMD-R12/MMD3 were significantly older than those with pseudometabolic or asymptomatic phenotypes (*p* < 0.05). The proportion of symptomatic patients increased as age advanced (Fig. 2). More than half of the cohort were symptomatic by their 4th decade of life, although there was substantial interindividual variability and some patients remained asymptomatic until late adulthood (Table 1, Fig. 1).

#### *Gender*

Gender-based analysis indicated that males predominated in LGMD-R12/MMD3 and asymptomatic phenotypes, while females presented mostly with pseudometabolic symptoms. Males tended to be younger than females (median age at last assessment 46 years [41;55] vs. 57 years [50;65]) (*p* < 0.05). Also, males exhibited significantly higher CK values (*p* < 0.05) (Table 1). Females had generally lower TLB scores and fewer affected muscles on MRI (supplementary Table S3). However, statistically significant differences in radiological features between males and females were not detected, except for a higher number of muscles with STIR hyperintensities in males (median [IQR] 4.0 [3.8;8.0] vs. 2.0 [0;3.0] in females) (p < 0.05). The small sample size and unequal gender distribution could have influenced this lack of significant differences.

#### *Genotype*

The presence of LOF variants (1 or 2) correlated with more severe phenotypes. All patients with LGMD-R12/MMD3 carried at least one LOF variant (100%) compared to 65% of those with pseudometabolic phenotype and 33% of asymptomatic individuals (*p* < *0.05).* Patients carrying LOF variants exhibited greater muscle involvement on MRI, reflected by higher TLB scores and the number of affected muscles, than those without LOF variants *(p* < *0.05).*

#### *Ancillary tests*

Regarding CK, only the minimum value showed a significant correlation across phenotypes, particularly distinguishing pseudometabolic patients from those with LGMD-R12/MMD3 (*p* < *0.05),* who had higher values. In contrast, maximum CK values did not differentiate between phenotypes.

Dystrophic features on muscle biopsy were mainly observed in patients with LGMD-R12/MMD3 (75%) and less frequently in those with the pseudometabolic phenotype (40%), while asymptomatic individuals showed mild myopathic findings (Table 2). Myopathic EMG patterns were present in all patients with LGMD-R12/MMD3 and in 55% and 45% of those with the pseudometabolic and asymptomatic phenotypes, respectively. However, neither biopsy nor EMG findings differed significantly between phenotypes.

#### *Phenotype variability between siblings*

**Fig. 4:**
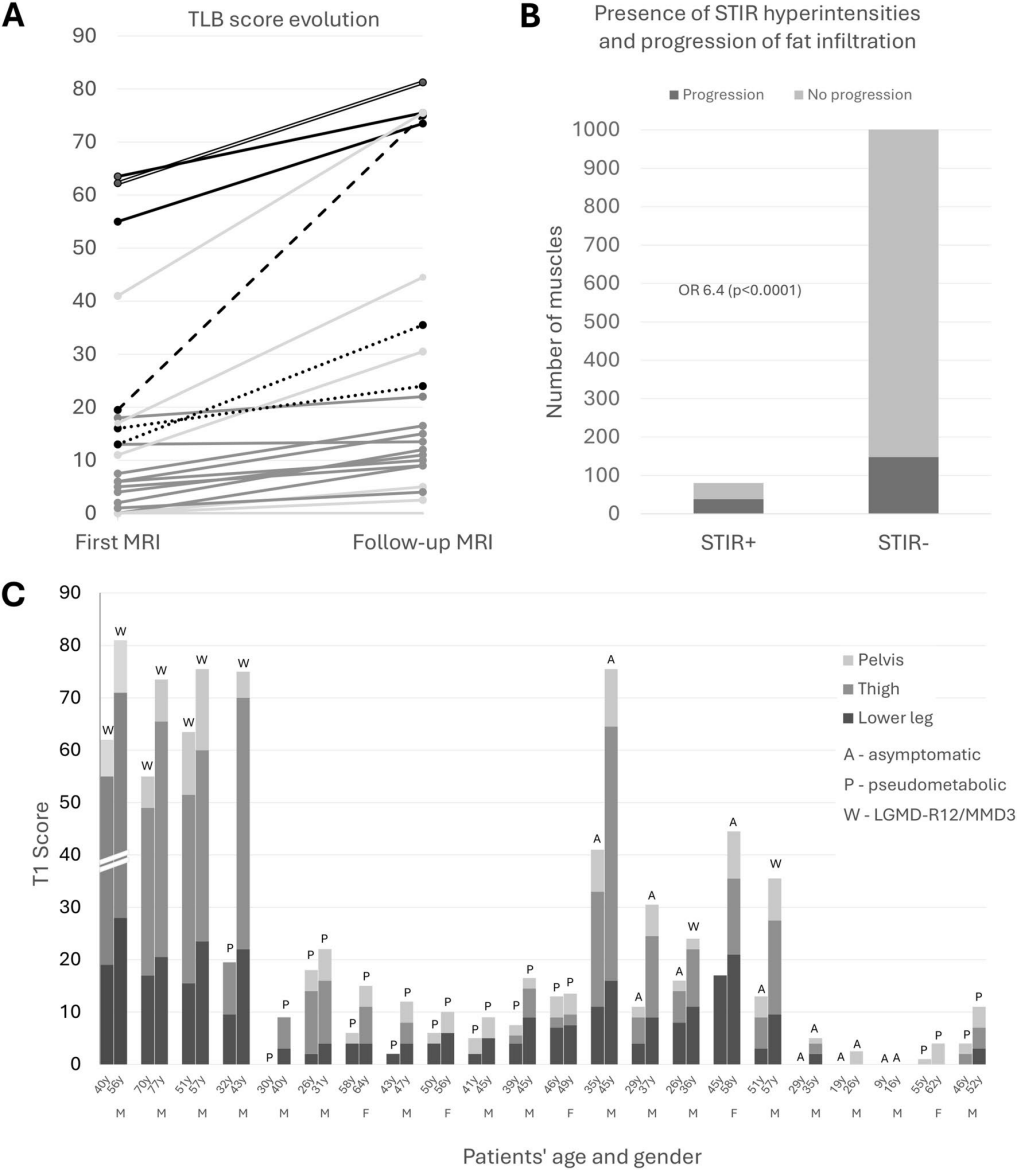
Progression of muscle involvement over time, as assessed by MRI. MRI scans from patient P05 demonstrate progression of muscle involvement over 11 years of follow-up (A–O). P05 exhibited a pseudometabolic phenotype at the first and second MRIs (ages 32 and 38). By the third MRI (age 43) he had developed mild disto-proximal weakness. At the first MRI, severe involvement of the gastrocnemius medialis (**A**) was observed along with mild fibrosis of the posterior thigh (**C**) and gluteus minimus (**E**); STIR hyperintensities affected the soleus (**B**) and biceps femoris (**D**), which later progressed to fat infiltration (**F**, **H**). At the second MRI, STIR hyperintensities persisted in the soleus (**G**) and newly appeared in the adductor magnus (**I**), evolving into severe fibrosis (**M**). By the third MRI, fat infiltration had progressed in the lower leg (K) and thighs (**M**); STIR hyperintensity appeared in the tibialis anterior (**L**), indicating ongoing active disease. Pelvic involvement remained restricted to the gluteus minimus (**J**, **O**), without detectable STIR hyperintensities (not shown). His brother, P06, who also exhibited pseudometabolic symptoms, showed mild fibrosis in GM (**P**), posterior thigh and adductors (**R**), with slight STIR hyperintensity restricted to GM (**Q**). Pelvic muscles were spared (**T**)

Phenotypic variability was observed among five pairs of siblings carrying identical genotypes (Table 2, Fig. 1, Table S1). In one pair, a 55-year-old male (P20) developed myalgia and weakness, whereas his 58-year-old sister (P19) remained asymptomatic despite showing a higher TLB score (44.5 vs. 35.5). Another pair, P10 (female) and her younger brother (P11), both with pseudometabolic symptoms, displayed similar MRI pattern and TLB scores (10 vs. 9) despite an 11-year age gap. Apart from these differences that could be gender-related, variability was also evident between male siblings: P05 developed disto-proximal weakness at 38 years, while his brother (P06) had only mild pseudometabolic symptoms at 40; also radiological involvement differed markedly (TLB score 75 vs. 9) as did mean CK levels (7579.5 vs. 2258.5 U/L) (Fig. 4). Additional pairs showed similar discordance: P07 experienced pseudometabolic symptoms at 24 years while his brother (P17) remained asymptomatic at 48; P02 developed weakness at 45 years whereas his brother (P01) did so at 24.

### Statistical analysis of multivariate model

We analyzed the correlation between phenotype severity (LGMD-R12/MMD3 > pseudometabolic > asymptomatic) and four variables: age, gender, presence of LOF variants, and TLB score. Increasing age, higher TLB score and LOF variants were associated with more severe phenotypes (OR = 1.377 per year; OR = 1.07 per point). By contrast, the effect of gender was uncertain (Table S4). The multivariate model was able to predict the LGMD-R12/MMD3 phenotype in 88% of cases, pseudometabolic phenotype in 72% and asymptomatic phenotype in 50%. In summary, older age, higher TLB score, and LOF variants were strongly associated with the LGMD-R12/MMD3 phenotype.

## Discussion

The present study integrates longitudinal clinical and radiological data from a well-characterized cohort of patients with *ANO5*-related muscle disorders, with long-term follow-up, thereby contributing to a better characterization of the natural history of this condition.

Our results demonstrate that, although clinically heterogeneous, *ANO5*-related myopathies share a common pattern of muscle involvement and follow a progressive course with variable rates of severity. These data support the concept that the spectrum of *ANO5*-related phenotypes, ranging from asymptomatic hyperCKemia to pseudometabolic and LGMD-R12/MMD3 phenotypes, represents a continuum rather than distinct entities, similar to dyspherlinopathies [24]. This overlap of manifestations, particularly between LGMD-R12 and MMD3, has been already suggested [2, 7, 11, 18]. Our findings further corroborate this, showing that most patients exhibit disto-proximal radiological muscle involvement, with gluteus minimus and GM affected in a similar proportion (86% and 83% of individuals, respectively), regardless of their phenotype.

Despite most patients exhibiting radiological changes, muscle weakness was detected in only 16% of the cohort at first assessment and over one-third were asymptomatic. This figure contrasts with previous LGMD-R12/MMD3-focused studies and data from a European Cohort of anoctaminopathies, in which 66–90% of patients presented the LGMD-R12/MMD3 phenotype [2, 9, 10]. Conversely, the proportion of asymptomatic individuals reported in other studies ranged from 36% [11] to 53% [12], data more consistent with our findings. While the precise contribution of anoctaminopathies to hyperCKemia remains unsettled, these findings support the inclusion of ANO5 variants in the diagnostic work-up of patients with elevated CK levels, in line with previous studies identifying ANO5 variants as a frequent cause of hyperCKemia [25]. Besides, in our recent paediatric hyperCKemia study, *ANO5* variants ranked third overall (after *DMD* and *CAPN3*) and were the leading cause in the juvenile subgroup, together with *DYSF* [26].

This is the first study to estimate the prevalence of *ANO5-*related disorders in the Spanish population, which appears comparable to other European cohorts [9, 11], although it is likely underestimated due to undiagnosed subclinical cases. Notably, the c.191dupA variant, reported as a founder mutation in Northern Europe [8] was the most frequent in our cohort, despite its different genetic background, suggesting either a widespread mutational event or a founder effect in our region, which warrants further investigation. However, current genetic databases report c.191dupA as very rare in Spain (CSVS AF 0.001, no homozygotes) [27], and less frequent than in other European populations (gnomAD NFE AF ~ 0.0021). Our findings, including five patients homozygous for c.191dupA, question this reported low incidence. This variant is situated within a seven-base adenosine homopolymer (AAAAAAA) and may elude detection in certain genetic tests, including some Next-Generation Sequencing (NGS) techniques. Specifically, duplications may be prone to being misidentified as sequencing artifacts or filtered out by automated bioinformatic pipelines that exclude small insertions/deletions in homopolymer regions. These factors likely result in a high false-negative rate in population databases and, consequently, prevent us from estimating the carrier frequency of c.191dupA in our dataset. 

This study underscores the progressive nature of the disease, further supported by MRI findings, although radiological changes did not always have a clinical impact. Notably, only 10% of patients experienced phenotype conversion during follow-up, whereas 80% exhibited progression of muscle involvement on MRI. We observed substantial subclinical muscle involvement, leading to overlapping MRI patterns across different clinical phenotypes, consistent with previous reports [17, 28]. Of note, asymptomatic individuals showed higher T1 scores and a greater number of affected muscles than those with the pseudometabolic phenotype, although these differences were not statistically significant. These observations suggest the influence of additional factors or mechanisms that may limit the detectable extent of muscle damage in pseudometabolic patients. They may also be related to the more balanced male-to-female ratio in this group (7:6) and to the higher susceptibility of males to develop the LGMD-R12/MMD3 phenotype, as previously described [2, 6, 12, 17, 28].

The distribution of involvement, and compensation by spared muscles, may explain the discrepancies between radiological findings and clinical manifestations in anoctaminopathies. Marked fatty infiltration in certain muscles, particularly those in the posterior compartment of the lower limbs, may remain clinically silent [14, 29]. In contrast, involvement of the gluteus maximus or gluteus medius is more likely to produce detectable weakness, as observed in our cohort.

This study also analyzed STIR hyperintensities as predictors of subsequent fat replacement, as reported in other muscular dystrophies[30, 31]. Muscles with STIR hyperintensities had over six-fold higher odds of developing or progressing fat replacement than those without. However, the detection of muscles with STIR hyperintensities without subsequent changes in their T1 score may reflect limitations of the Mercuri scale, unable to capture subtle or slow disease progression, a limitation that could be overcome using quantitative MRI techniques. Also, the potential influence of protective modifiers delaying structural damage cannot be excluded. Nevertheless, STIR hyperintensities remain a robust indicator of ongoing muscle pathology and a useful predictor of future degeneration.

Our study aimed to investigate factors influencing the phenotypic variability and disease progression, with patient age emerging as one of the most important. The proportion of asymptomatic individuals decreased with age, remaining above 80% before the 4th decade and falling below 20% after the 6th. Older age also correlated with higher TLB scores and a greater number of muscles affected on MRI. However, we observed substantial inter-individual variability, laying proof of additional disease modifiers that warrant further investigation.

There is growing evidence that individuals carrying LOF variants in *ANO5* exhibit more severe disease [2]. Our findings support this observation, as patients carrying at least one LOF variant had more pronounced clinical and MRI involvement. Nevertheless, identical genotypes resulted in variable presentations and disease course, as illustrated by the differences observed among siblings. Such variability likely reflects additional, yet unidentified, modifying factors. Gender also influenced clinical presentation. Females, underrepresented in the cohort, were generally older and less severely affected, consistent with prior reports suggesting that milder disease in women may contribute to underdiagnosis [7, 32]. This gender imbalance likely contributed to the lack of statistical significance between males and females for most analyzed variables.

Regarding complementary tests, dystrophic features on muscle biopsy and myopathic changes on EMG tended to correlate with symptomatic phenotypes, although these associations did not reach statistical significance. Minimum CK values demonstrated greater discriminatory power between phenotypes compared to peak values.

Our findings further support that cardiac and respiratory involvement is rare in *ANO-5* myopathies [2–4]. Specifically, cardiological alterations detected in a small subset of patients were mild, non-progressive, and did not require intervention.

This study has several limitations. The sample size may limit statistical power analyses and extrapolation, while the single-centre design involving patients with similar demographic and genetic backgrounds could introduce a founder-effect bias. Muscle fat infiltration was evaluated using a semiquantitative scale, which is subject to interobserver variability and may not detect subtle changes that fall within the same score. Finally, as data were collected during routine clinical practice, functional tests were not consistently performed, which could have enhanced the clinical assessment.

To address these limitations, future research should prioritize prospective, multi-centre studies involving broader populations and incorporating diverse outcome measures. Such efforts will be crucial for identifying additional disease modifiers and potential therapeutic targets, ultimately improving care for patients with *ANO5*-related myopathies.

## Conclusion

*ANO5*-related myopathies are age-dependent, slowly progressive disorders characterized by broad phenotypic variability and a consistent radiological pattern. Muscle MRI is a valuable tool for detecting early changes and monitoring disease progression. STIR hyperintensities indicate active disease and may predict future muscle degeneration. As therapeutic development advances, sensitive outcome measures, including advanced imaging and wearable technologies, will be critical for evaluating interventions and optimizing patient care.

## Supplementary Information

Below is the link to the electronic supplementary material.

Supplementary file1 (DOCX 52 KB)

## Data Availability

The data that support the findings of this study are available from the corresponding author upon reasonable request.
