# Supplementary material for: Long-term clinical and radiological trajectories in ANO5-related myopathies highlight muscle MRI as a predictor of disease progression
Source: J Neurol. 2026 Apr 15;273(5):270. doi: 10.1007/s00415-026-13805-1 (PMC13079537; doi:10.1007/s00415-026-13805-1)
Supplement: Supplementary file 1 — Supplementary file1 (DOCX 52 KB) [file 415_2026_13805_MOESM1_ESM.docx]

| Variant in *a ANO5* (NM_213599.3) | **P01- P02** P08 P12 P15 | **P05-P06 P10-P11** P16 | P03 | P04 | **P07-P17** | P09, P21 | P13 | P14 | P18 | **P19- P20** | P22 | P23 | P24 | P25 | P26 | P27 | P28 | P29 | P30 | % | Variant functional type | ACMG variant classification |
| --- | --- | --- | --- | --- | --- | --- | --- | --- | --- | --- | --- | --- | --- | --- | --- | --- | --- | --- | --- | --- | --- | --- |
| c.191dup p.(Asn64LysfsTer15) | x | xx |  |  |  |  |  | x | x | x |  |  |  |  | x |  |  | x |  | 35 | Frameshift | Pathogenic |
| c.692G>T p.(Gly231Val) | x |  |  |  | x | x | x |  |  |  | x |  |  |  |  | xx |  |  |  | 22 | Missense | Likely pathogenic |
| c.172C>T p.(Arg58Trp) |  |  |  |  |  | x |  |  |  |  |  |  | x | xx |  |  |  |  | x | 10 | Missense | Pathogenic |
| c.653A>G p.(Tyr218Cys)* |  |  |  |  |  |  |  |  |  |  |  | xx |  |  |  |  |  |  | x | 5 | Missense | VUS |
| c.950C>A p.(Ala317Glu)* |  |  |  |  | x |  |  |  |  |  |  |  |  |  |  |  |  |  |  | 3 | Missense | VUS |
| c.2012A>G p.(Tyr671Cys) |  |  |  |  |  |  |  |  |  | x |  |  |  |  |  |  |  |  |  | 3 | Missense | Likely pathogenic |
| c.1627dup p.(Met543AsnfsTer11) |  |  | xx |  |  |  |  |  |  |  |  |  |  |  |  |  |  |  |  | 3 | Frameshift | Pathogenic |
| c.2339_2349del p.(Ser780IlefsTer5)* |  |  |  | x |  |  | x |  |  |  |  |  |  |  |  |  |  |  |  | 3 | Frameshift | Likely pathogenic |
| c.155A>G p.(Asn52Ser)^ |  |  |  |  |  |  |  |  |  |  |  |  | x |  |  |  |  | x |  | 3 | Missense | VUS |
| c.2222C>G p.(Ser741Cys)* |  |  |  |  |  |  |  | x |  |  |  |  |  |  |  |  | x |  |  | 3 | Missense | Likely pathogenic |
| c.108_109del p.(Glu36AspfsTer7) |  |  |  |  |  |  |  |  | x |  |  |  |  |  |  |  |  |  |  | 2 | Frameshift | Pathogenic |
| c.148C>T p.(Arg50Ter) |  |  |  | x |  |  |  |  |  |  |  |  |  |  |  |  |  |  |  | 2 | Nonsense | Pathogenic |
| c.1898+1G>A p.? |  |  |  |  |  |  |  |  |  |  | x |  |  |  |  |  |  |  |  | 2 | Intronic | Pathogenic |
| c.1210C>T p.(Arg404Ter) |  |  |  |  |  |  |  |  |  |  |  |  |  |  | x |  |  |  |  | 2 | Nonsense | Pathogenic |
| c.2317A>G p.(Met773Val)* |  |  |  |  |  |  |  |  |  |  |  |  |  |  |  |  | x |  |  | 2 | Missense | VUS |

Table S1. Genetic variants identified in the cohort

Variants identified in the cohort. *novel variants; ^variant reported with conflictive significance; pairs of siblings are marked in bold: P01-02; P05-06; P07-17; P10-11; P19-20; P08 and P18 were aunt and nephew and P04 andP13 were father and daughter.

Table S2. MRI assessments and radiological data of the cohort

|  | P01 | P03 | P04 | P05 | P06 | P07 | P08 | P09 | P10 | P11 | P12 | P13 | P14 | P15 | P16 | P17 | P18 | P19 | P20 | P21 | P22 | P23 | P24 | P25 | P26 | P27 | P28 | P29 | P30 |
| --- | --- | --- | --- | --- | --- | --- | --- | --- | --- | --- | --- | --- | --- | --- | --- | --- | --- | --- | --- | --- | --- | --- | --- | --- | --- | --- | --- | --- | --- |
| Gender | M | M | M | M | M | M | F | M | F | M | M | F | F | M | M | M | M | F | M | M | M | F | M | M | M | F | F | F | M |
| **Age MRI_1** | **40** | **70** | **51** | **32** | **30** | **26** | **58** | **43** | **50** | **41** | **39** | **31** | **46** | **35** | **29** | **47** | **26** | **45** | **51** | **29** | **19** | **49** | **9** | **52** | **38** | **45** | **55** | **78** | **46** |
| Phenotype_MRI1 | W | W | W | P | P | P | P | P | P | P | P | P | P | A | A | A | A | A | A | A | A | A | A | A | P | P | P | W | P |
| Lower leg_T1 score MRI1 | 38 | 17 | 15,5 | 9,5 | 0 | 2 | 4 | 2 | 4 | 2 | 4 | 6 | 7 | 11 | 4 | 6 | 8 | 17 | 3 | 0 | 0 | 11,5 | 0 | 20 | 6 | 0 | 0 | 14 | 0 |
| Thigh_T1 score MRI1 | 72,5 | 32 | 36 | 10 | 0 | 12 | 0 | 0 | 0 | 0 | 1,5 | 0 | 2 | 22 | 5 | 2 | 6 | 0 | 6 | 0 | 0 | 13 | 0 | 54,5 | 0 | 0 | 0 | 40 | 2 |
| Pelvis_T1 score MRI1 | 14 | 6 | 12 | 0 | 0 | 4 | 2 | 0 | 2 | 3 | 2 | 2 | 4 | 8 | 2 | 4 | 2 | 0 | 4 | 0 | 0 | 7 | 0 | NA | 0 | 0 | 1 | 14 | 2 |
| TLB_T1 score MRI1 | 124,5 | 55 | 63,5 | 19,5 | 0 | 18 | 6 | 2 | 6 | 5 | 7,5 | 8 | 13 | 41 | 11 | 12 | 16 | 17 | 13 | 0 | 0 | 31,5 | 0 | 74,5 | 6 | 0 | 1 | 68 | 4 |
| Axial involvement MRI1 | X | - | X | - | - | - | - | - | - | - | - | - | X | - | - | - | - | - | - | - | - | X | - | - | - | - | - | X | - |
| STIR hyperintensity MRI1 | ++ | ++ | + | + | - | + | + | + | + | + | + | + | + | ++ | ++ | + | + | - | + | + | - | + | + | NA | - | - | + | - | + |
| Lower leg muscles affected at MRI1, n | 15 | 8 | 6 | 3 | 0 | 2 | 4 | 2 | 2 | 2 | 4 | 4 | 2 | 4 | 2 | 4 | 4 | 5 | 2 | 0 | 0 | 6 | 0 | 3 | 4 | 0 | 0 | 6 | 0 |
| Thigh muscles affected at MRI1, n | 20 | 16 | 16 | 6 | 0 | 12 | 0 | 0 | 0 | 0 | 1 | 0 | 2 | 12 | 5 | 2 | 6 | 0 | 6 | 0 | 0 | 6 | 0 | 9 | 0 | 0 | 0 | 16 | 1 |
| Pelvis muscles affected at MRI1, n | 6 | 4 | 6 | 0 | 0 | 2 | 2 | 0 | 2 | 2 | 2 | 2 | 2 | 2 | 2 | 2 | 2 | 0 | 2 | 0 | - | 4 | 0 | NA | 0 | 0 | 2 | 5 | 2 |
| Total lower body muscles affected at MRI1, n | 41 | 28 | 28 | 9 | 0 | 16 | 6 | 2 | 4 | 4 | 7 | 6 | 6 | 18 | 9 | 8 | 12 | 5 | 10 | 0 | 0 | 16 | 0 | 12 | 4 | 0 | 2 | 27 | 3 |
| **Age MRI_2** | **56** | **77** | **57** | **43** | **40** | **31** | **64** | **47** | **56** | **45** | **45** | **-** | **49** | **45** | **37** | **-** | **36** | **58** | **57** | **35** | **26** | **-** | **16** | **-** | **-** | **-** | **62** | **-** | **52** |
| **Years MRI interval** | **16** | **7** | **6** | **11** | **10** | **5** | **6** | **4** | **6** | **4** | **6** | **-** | **3** | **10** | **8** | **-** | **10** | **13** | **6** | **6** | **7** | **-** | **7** | **-** | **-** | **-** | **7** | **-** | **6** |
| Phenotype _MRI2 | W | W | W | W | P | P | P | P | P | P | P | - | P | A | A | - | W | A | W | A | A | - | A | - | - | - | P | - | P |
| Lower leg_T1 score MRI2 | 56 | 20,5 | 23,5 | 22 | 3 | 4 | 4 | 4 | 6 | 5 | 9 | - | 7,5 | 16 | 9 | - | 11 | 21 | 9,5 | 2 | 0 | - | 0 | - | - | - | 0 | - | 3 |
| Thigh_T1 score MRI2 | 86,5 | 45 | 36,5 | 48 | 6 | 12 | 7 | 4 | 0 | 0 | 5,5 | - | 2 | 48,5 | 15,5 | - | 11 | 14,5 | 18 | 2 | 0 | - | 0 | - | - | - | 0 | - | 4 |
| Pelvis_T1 score MRI2 | 20 | 8 | 15,5 | 5 | 0 | 6 | 4 | 4 | 4 | 4 | 2 | - | 4 | 11 | 6 | - | 2 | 9 | 8 | 1 | 2,5 | - | 0 | - | - | - | 4 | - | 4 |
| TLB_T1 score MRI2 | 162,5 | 73,5 | 75,5 | 75 | 9 | 22 | 15 | 12 | 10 | 9 | 16,5 | - | 13,5 | 75,5 | 30,5 | - | 24 | 44,5 | 35,5 | 5 | 2,5 | - | 0 | - | - | - | 4 | - | 11 |
| TLB_T1 score difference MRI2/MRI1 | 38 | 18,5 | 12 | 55,5 | 9 | 4 | 9 | 10 | 4 | 4 | 9 | - | 0,5 | 34,5 | 19,5 | - | 8 | 27,5 | 22,5 | 5 | NA | - | 0 | - | - | - | 3 | - | 7 |
| TLB_T1 score change/year | 2,4 | 2,6 | 2,0 | 5,0 | 0,9 | 0,8 | 1,5 | 2,5 | 0,7 | 1,0 | 1,5 | - | 0,2 | 3,5 | 2,4 | - | 0,8 | 2,1 | 3,8 | 0,8 | - | - | 0,0 | - | - | - | 0,4 | - | 1,2 |
| Axial involvement MRI2 | X | X | - | X | - | - | - | - | - | - | - | - | X | X | - | - | - | X | - | X | X | - | - | - | - | - | X | - | - |
| STIR hyperintensity MRI2 | ++ | ++ | ++ | + | + | ++ | - | ++ | + | + | + | - | ++ | ++ | ++ | - | ++ | + | ++ | + | + | - | + | - | - | - | + | - | - |
| Lower leg muscles affected at MRI2, n | 20 | 8 | 11 | 8 | 3 | 2 | 4 | 2 | 2 | 3 | 5 | - | 2 | 4 | 3 | - | 6 | 6 | 4 | 2 | 0 | - | 0 | - | - | - | 0 | - | 3 |
| Thigh muscles affected at MRI2, n | 24 | 18 | 16 | 20 | 6 | 12 | 6 | 2 | 0 | 0 | 4 | - | 2 | 14 | 8 | - | 8 | 8 | 9 | 2 | 0 | - | 0 | - | - | - | 0 | - | 2 |
| Pelvis muscles affected at MRI2, n | 6 | 4 | 6 | 2 | 0 | 4 | 2 | 2 | 2 | 2 | 2 | - | 2 | 4 | 4 | - | 2 | 4 | 4 | 1 | 2 | - | 0 | - | - | - | 2 | - | 2 |
| Total lower body muscles affected at MRI2, n | 50 | 30 | 33 | 30 | 9 | 18 | 12 | 6 | 4 | 5 | 11 | - | 6 | 22 | 15 | - | 16 | 18 | 17 | 5 | 2 | - | 0 | - | - | - | 2 | - | 7 |
| Difference of affected muscles MRI2/MRI1, n | 9 | 2 | 5 | 21 | 9 | 2 | 6 | 4 | 0 | 1 | 4 | - | 0 | 4 | 6 | - | 4 | 13 | 7 | 5 | 2 | - | 0 | - | - | - | 0 | - | 4 |
| Visual progression of radiological involvement | M | M | M | S | S | S | S | M | M | S | M | - | M | M | M | - | M | S | M | S | S | - | S | - | - | - | S | - | S |

MRI_1: first MRI, Phenotype: W- LGMD-R12/MMD3, P-Paucisymptomatic, A-asymptomatic; TLB – total lower body; Axial – paraspinal muscles involvement; STIR hyperintensities: ++ (marked), + (slight), - (none); No.mm.– number of muscles affected; MRI_2: follow-up MRI; TLB score change/year: the annual rate of progression of fibrosis was calculated as the difference between these two parameters calculated in last and first MRIs divided by the time interval in years between scans[TLB/year: ΔTLB(last–first MRI) ÷ Δtime (years)]; overall radiological progression: M – marked, S – subtle.

Table S3. MRI analysis of patients grouped by phenotype, gender and genotype at baseline and follow-up imaging

| **Variable** | **Phenotype** | | | | | | | | **Gender** | | | **Genotype (LOF)** | | |
| --- | --- | --- | --- | --- | --- | --- | --- | --- | --- | --- | --- | --- | --- | --- |
|  | LGMD-R12/MMD3 | W vs. A | Asympto-matic | A vs. P | Pseudo-metabolic | P vs.W | All | W vs. AP | Females (n=9) | Males (n=21) | *p* | No LOF (n=11) | ≥1 LOF (n=19) | *p* |
| **T1- score at first MRI** | | | | | | | | | | | | | | |
| Lower leg | 17.8 [15.1–24.5] | * | 6.0 [1.5–11.2] | *ns* | 3.0 [0.5–5.5] | ** | 4.0 [2.0;11.0] | ** | 6.0 [4.0;11.5] | 4.0 [1.5;9.9] | ns | 0 [0;4.0] | 6.5 [4.0;13.2] | * |
| Thigh | 38.0 [35.0–48.1] | * | 5.0 [0–9.5] | *ns* | 0 [0–1.9] | ** | 2.0 [0;12.0] | ** | 0 [0;2.0] | 3.5 [0;14.5] | ns | 0 [0;7.0] | 3.5 [0;19.0] | ns |
| Pelvis | 13.0 [10.5–14.0] | * | 2.0 [0–4.0] | *ns* | 2.0 [0–2.0] | ** | 2.0 [0;4.0] | ** | 2.0 [2.0;4.0] | 2.0 [0;4.0] | ns | 0 [0;3.0] | 2.0 [2.0;5.5] | ns |
| TLB | 65.8 [ 62.1;82.1] | * | 13.0 [5.5;24.2] | *ns* | 6.0 [2.5;7.9] | ** | 11.0 [4.0;19.5] | ** | 8.0 [6.0;17.0] | 11.5 [3.5;24.9] | ns | 2.0 [0;15.0] | 13.0 [6.4;35.6] | * |
| **Number of muscles affected at first MRI** | | | | | | | | | | | | | | |
| Lower leg | 7.0 [6.0–9.8] | * | 4.0 [1.0–4.5] | *ns* | 2.0 [0.5–3.8] | ** | 3.0 [2.0;4.0] | ** | 4.0 [2.0;5.0] | 2.5 [1.5;4.0] | ns | 0 [0;3.0] | 4.0 [2.0;4.8] | * |
| Thigh | 16.0 [16.0–17.0] | *ns* | 5.0 [0–6.0] | *ns* | 0 [0–1.0] | ** | 1.0 [0;6.0] | ** | 0 [0;2.0] | 3.5 [0;12.0] | ns | 0 [0;4.0] | 3.5 [0;10.5] | ns |
| Pelvis | 5.5 [4.8–6.0] | ** | 2.0 [0–2.0] | *ns* | 2.0 [0–2.0] | ** | 2.0 [0;2.0] | ** | 2.0 [2.0;2.0] | 2.0 [0;2.0] | ns | 0 [0;2.0] | 2.0 [2.0;2.0] | ns |
| Total | 28.0 [27.8–31.2] | * | 9.0 [2.5–14.0] | *ns* | 4.0 [2.2–6.0] | ** | 6.0 [3.0;16.0] | ** | 6.0 [4.0;6.0] | 8.5 [2.8;16.5] | ns | 2.0 [0;12.0] | 8.0 [5.2; 16.5] | ns |
| **T1- score at follow-up MRI** | | | | | | | | | | | | | | |
| Lower leg | 20.5 [15.2;23.1] | *ns* | 9.0 [2.0;16.0] | *ns* | 4.5 [3.2;6.0] | ** | 6.0 [4.0;14.0] | ** | 6.0 [4.0;11.5] | 7.5 [3.8;16.8] | ns | 3.0 [0;5.0] | 9.2 [6.0;18.2] | ** |
| Thigh | 42.5 [37.4;47.2] | * | 13.0 [2.0;15.5] | *ns* | 3.0 [0;5.9] | ** | 6.0 [0;18.0] | ** | 2.0 [0;13.0] | 8.5 [2.0;38.6] | ns | 2.0 [0;8.0] | 12.8 [2.9;39.1] | ns |
| Pelvis | 11.0 [8.0;15.1] | * | 4.0 [1.0;7.0] | *ns* | 4.0 [2.0;4.0] | ** | 4.0 [2.0;7.0] | ** | 4.0 [4.0;7.0] | 4.0 [1.8;6.5] | ns | 4.0 [0.5;4.0] | 4.5 [2.5;8.8] | ns |
| TLB | 73.5 [69.0;75.4] | * | 30.5 [5.0;44.5] | *ns* | 10.5 [8.2;14.6] | ** | 15.0 [9.0;44.5] | ** | 13.5 [8.0;31.5] | 19.2 [9.0;72.6] | ns | 11.0 [3.2;17.0] | 27.2 [10.9;71.0] | * |
| **Number of muscles affected at follow-up MRI** | | | | | | | | | | | | | | |
| Lower leg | 8.0 [6.5;10.2] | * | 4.0 [2.0;6.0] | *ns* | 3.0 [2.0;4.0] | ** | 4.0 [2.0;6.0] | ** | 4.0 [2.0;6.0] | 4.0 [2.8;6.2] | ns | 2.0 [0;3.5] | 4.5 [3.2;6.8] | ** |
| Thigh | 17.0 [16.0;19.5] | *** | 6.0 [2.0;8.0] | *ns* | 2.0 [0;5.5] | ** | 6.0 [0;12.0] | ** | 2.0 [0;6.0] | 7.0 [2.0;16.0] | ns | 2.0 [0;4.0] | 8.0 [2.5;16.0] | ns |
| Pelvis | 4.5 [4.0;5.8] | *ns* | 2.0 [1.0;4.0] | *ns* | 2.0 [2.0;2.0] | ** | 2.0 [2.0;4.0] | ** | 2.0 [2.0;4.0] | 2.0 [1.8;4.0] | ns | 2.0 [0.5;2.0] | 2.0 [2.0;4.0] | ns |
| Total | 30.0 [27.8;32.2] | * | 15.0 [5.0;18.0] | *ns* | 6.0 [4.2;10.5] | ** | 11.0 [5.0;18.0] | ** | 6.0 [4.0;16.0] | 13.0 [5.8;27.2] | ns | 6.0 [2.0;12.0] | 16.0 [6.8;27.0] | * |
| **Annual progression rate** | | | | | | | | | | | | | | |
| TLB | 2.0 [2.0;2.4] | *ns* | 1.1 [0.4;1.9] | *ns* | 0.8 [0.7;1.3] | *ns* | 1.2 [ 0.8; 2.2] | *ns* | 0.7 [0.3; 1.5] | 1.3 [0.8; 2.2] | ns | 0.8 [0.2; 1.0] | 1.9 [0.9; 2.2] | ns |
| Total no. muscles | 0.6 [0.6;0.8] | *ns* | 0.6 [0.2;0.8] | *ns* | 0.5 [0.1;0.9] | *ns* | 0.6 [0.3; 0.8] | *ns* | 0 [0;1.0] | 0.6 [0.4;0.8] | ns | 0.4 [0.1;0.8] | 0.6 [0.3;0.9] | ns |

Values are presented in median and inter-quartile range [IQR]. T1-score correspond to the sum of Mercuri scores across all muscles in each compartment (lower leg, thigh, pelvis), and the total lower body score (TLB) as the sum of the three compartments; The number of muscles affected, The annual progression rate was calculated as a difference between TLB at last available and first MRI divided by years span between MRIs; Statistical difference was calculated for following groups: W vs. A – LGMD/MMD3 vs. asymptomatic, A vs. P – asymptomatic vs. pseudometabolic, P vs. W – pseudometabolic vs. LGMD-R12/MMD3, W vs. AP – LGMD-R12/MMD3 vs. asymptomatic and pseudometabolic together; females vs. males; individuals with absence or presence of loss-of-function genetic variants (LOF) as “No LOF” vs. “>=1 LOF” (at least one LOF variant); P values are given as * = p<0.05, ** = p<0.01, ns – non-significant, p>0.05

Table S4. Posterior estimates of the Bayesian ordinal mixed-effects regression model.

| Predictor | β (Estimate) | SE | 95% CrI | P(β>0) | OR (exp(β)) |
| --- | --- | --- | --- | --- | --- |
| Intercept [1] | 18.05 | 10.65 | [3.71, 45.23] | – | – |
| Intercept [2] | 27.25 | 12.70 | [10.34, 59.67] | – | – |
| Gender (Male) | 2.33 | 3.73 | [-4.61, 10.45] | 0.757 | 10.28 |
| LOF variants (Yes) | 4.42 | 3.56 | [-1.53, 11.98] | 0.931 | 83.10 |
| Age | 0.32 | 0.19 | [0.07, 0.81] | 0.994 | 1.38 |
| TLB score | 0.07 | 0.06 | [-0.03, 0.22] | 0.920 | 1.07 |
| Random intercepts | 6.32 | 2.74 | [2.76, 13.29] | – | – |

SE = posterior standard error; CrI = Bayesian 95% credible interval; OR = odds ratio. Intercept[1] and Intercept[2] are threshold parameters separating the ordinal outcome categories (asymptomatic vs. pseudometabolic/LGMD-R12/MMD3, and asymptomatic/pseudometabolic vs. LGMD-R12/MMD3, respectively). These thresholds define cut-points in the latent scale and are not interpreted as clinical effects.
